# PAS Kinase: A Nutrient and Energy Sensor “Master Key” in the Response to Fasting/Feeding Conditions

**DOI:** 10.3389/fendo.2020.594053

**Published:** 2020-12-18

**Authors:** Verónica Hurtado-Carneiro, Ana Pérez-García, Elvira Alvarez, Carmen Sanz

**Affiliations:** ^1^ Department of Biochemistry and Molecular Biology, Faculty of Medicine, Complutense University of Madrid, Institute of Medical Research at the Hospital Clínico San Carlos (IdISSC), Ciudad Universitaria, Madrid, Spain; ^2^ Department of Physiology, Faculty of Medicine, Complutense University of Madrid, Madrid, Spain; ^3^ Department of Cell Biology, Faculty of Medicine, Complutense University of Madrid, Madrid, Spain

**Keywords:** food intake, metabolic sensors, mTOR/S6K, Per-Arnt-Sim kinase, obesity, diabetes, glucose, high-fat diet

## Abstract

The protein kinase with PAS domains (PASK) is a nutrient and energy sensor located in the cells of multiple organs. Many of the recent findings for understanding PASK functions in mammals have been reported in studies involving PASK-deficient mice. This minireview summarizes the PASK role in the control of fasting and feeding responses, focusing especially on the hypothalamus and liver. In 2013, PASK was identified in the hypothalamic areas involved in feeding behavior, and its expression was regulated under fasting/refeeding conditions. Furthermore, it plays a role in coordinating the activation/inactivation of the hypothalamic energy sensors AMPK and mTOR/S6K1 pathways in response to fasting. On the other hand, PASK deficiency prevents the development of obesity and non-alcoholic fatty liver in mice fed with a high-fat diet. This protection is explained by the re-establishment of several high-fat diet metabolic alterations produced in the expression of hepatic transcription factors and key enzymes that control the main metabolic pathways involved in maintaining metabolic homeostasis in fasting/feeding responses. This minireview covers the effects of PASK inactivation in the expression of certain transcription factors and target enzymes in several metabolic pathways under situations such as fasting and feeding with either a standard or a high-fat diet.

## Introduction

Nutrient sensors are molecules that detect nutritional changes and coordinate adaptation responses to them in order to maintain cellular metabolic and energy homeostasis. The Per-Arnt-Sim (PAS) kinase (PASK) protein, also termed PASKIN, is a metabolic sensor that has been preserved during the evolution from yeast to human. PASK has been described as a regulator of several metabolic and energy processes (1–3). Our main understanding of PASK functions in mammals began with the inactivation of PASK in mice in 2003 (4). Initial studies showed that PASK-deficient mice had no developmental, growth or reproductive defects (4). The first and most revealing data on the importance of PASK were forthcoming when PASK-deficient mice were subjected to a high-fat diet (HFD) (5). This led to the study of how PASK deficiency protects against the development of obesity brought on by HFDs, being partly due to a high metabolic rate in skeletal muscle. In addition, the role of PASK in insulin and glucagon secretion has already been described (6, 7). This review contains the most recent evidence and advances made involving the mechanisms that allow PASK to play a decisive role in the control of multiple cellular functions particularly related to cellular and organ response to fasting/feeding adaptation.

## Pask Expression and Function in Hypothalamic Areas

In 2013, PASK was identified in the hypothalamic areas involved in feeding behavior, and its expression was regulated under fasting/refeeding conditions (8, 9). Both the ventromedial hypothalamus (VMH) and the lateral hypothalamus (LH) areas were referred to as the centers of satiety and hunger, respectively (10). They play opposite functions in regulating food intake. The PASK expression is regulated *in vivo* in response to fasting/refeeding conditions. This effect is clearer in the LH: PASK-coding mRNAs are reduced by fasting and increased by refeeding conditions (9). The effect observed after refeeding *in vivo* is the opposite to the glucose effect found in the VMH and LH in hypothalamic organotypic cultures and neuroblastoma N2A cells. However, the effect is similar to those produced in the presence of both glucose and the anorexigenic glucagon-like peptide-1 (GLP-1) - an incretin release from intestinal L-cells in response to feeding (11–13). This suggests that the changes observed in PASK expression in response to fasting/refeeding *in vivo* are the sum of the effects of glucose and hormonal levels.

Hypothalamic metabolic sensors play an important role in the control of feeding and energy homeostasis. They respond to changes in nutrients and to orexigenic peptides such as ghrelin (14, 15), or anorexigenic ones such as leptin (14, 16), and their activation/inhibition regulates hunger or satiety responses, and therefore food intake. For example, the hypothalamic AMP-activated protein kinase (AMPK) is activated by fasting and inhibited by refeeding (17–19). Furthermore, most orexigenic signals, such as ghrelin (14, 20, 21), adiponectin (22) and cannabinoids (23) activate AMPK. By contrast, AMPK is inhibited by anorexigenic signals, such as leptin (14, 17), GLP-1 (24), estradiol (25, 26), and insulin (17, 27). Other hypothalamic sensors involved in the control of feeding and the regulation of energy balance are the pathway of the mammalian target of rapamycin (mTOR) and S6 kinase (S6K). This pathway is activated by glucose and amino acids, inhibiting food intake (16, 19, 28).

**Figure 1:**
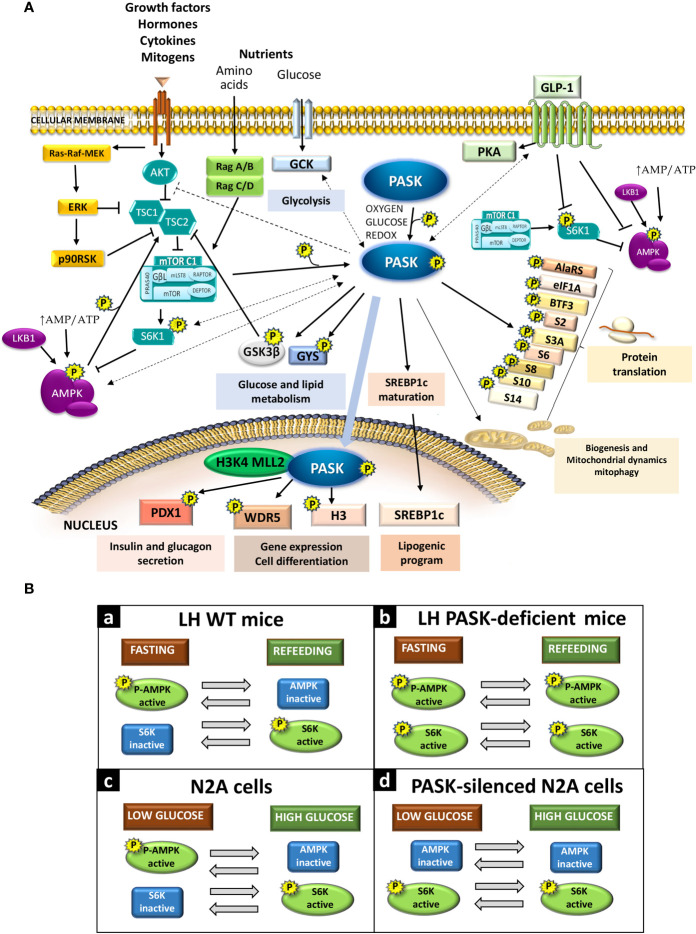
**(A)** Per-Arnt-Sim kinase (PASK) signaling interaction with other metabolic pathways. PASK substrates and selected physiological effects due to activation or inhibition (direct: solid lines; indirect: dashed lines) of certain signaling pathways. **(B)** PASK function in the activation of AMPK and S6K in response to glucose availability. Panels (a) and (b)*: In vivo* AMPK and S6K activities under fasting or refeeding conditions in the LH in WT mice and PASK-deficient mice, respectively. Panels (c) and (d): Response of AMPK and S6K activities to low or high glucose in neuroblastoma N2A cells or PASK-silenced N2A cells.

The activities of both pathways (AMPK and mTOR/S6K) are reciprocally coordinated and inversely regulated (i.e., when AMPK is activated, the mTOR/S6K pathway stops, and vice versa), ensuring the precise adjustment of intake. AMPK thereby phosphorylates the tuberous sclerosis complex 2 (TSC2), inducing mTORC1 inhibition mediated by the TSC1-TSC2 complex (29). AMPK also phosphorylates the rapamycin-sensitive adaptor protein of mTOR (RAPTOR) in mTORC1, prompting this complex’s downregulation (30). Likewise, S6K1 phosphorylates and inhibits AMPKα2 in the hypothalamus during the activation of the mTOR/S6K1 pathway (31) (**Figure 1A**).

PASK plays a role in coordinating the regulation in response to the activation/inactivation of the hypothalamic AMPK and mTOR/S6K1 pathways. PASK-deficiency impairs the coordination of the AMPK and mTOR/S6K1 pathways, which means both pathways are activated at the same time under fasting and feeding conditions (9). The inhibition of mTOR/S6K through AMPK activation requires the coordinated phosphorylation of TSC2 by glycogen synthase kinase 3β (GSK3β) (29), which is a PASK substrate *in vitro* (**Figure 1A**). PASK deficiency could therefore alter hypothalamic GSK3β activity (32).

The relationship between PASK deficiency and the impaired response of the AMPK and mTOR/S6K pathways has been confirmed in PASK-silenced N2A cells (8). PASK knockdown N2A cells under low glucose concentrations have higher ATP content, a lack of stimulation of AMPK, and enhanced activation of S6K. Schematic diagrams are provided of the activation degree of the AMPK and mTOR/S6K pathways under fasting and refeeding conditions in control wild-type (WT) mice [**Figure 1B (a)**] and PASK-deficient mice [**Figure 1B (b)**] and in PASK-silenced cells [**Figure 1B (d)**] or control N2A cells [**Figure 1B (c)**] under low or high glucose concentrations.

The deregulation of these signaling pathways could induce eating disorders that may lead to other pathologies such as obesity and type 2 diabetes. The hypothalamic PASK function could be decisive for maintaining nutritional and energy homeostasis. Modified responses of hypothalamic AMPK activity have been previously correlated to obesity and type 2 diabetes (33). The use of a specific AMPKα2-knockout in both neuronal populations, expressing orexigenic and anorexigenic peptides with opposite effects on food intake, modifies feeding behavior and energy and body weight homeostasis (34, 35). The AMPKα2-knockout in neuronal populations expressing orexigenic peptides therefore develops an age-dependent lean phenotype (34), while the AMPKα2-knockout in the neuronal population expressing anorexigenic peptides leads to obesity (34). In turn, the AMPKβ1-knockout mice recorded reduced food intake and total body weight (36) while AMPKβ2 deletion develops hyperinsulinemia and glucose intolerance under an HFD (37).

The mTOR/S6K hypothalamic pathway has also been related to orexigenic or anorexigenic responses (16, 28, 38–40). TSC1-deficient mice in the neuronal population expressing anorexigenic peptides, which promote increased mTOR activation, have developed an orexigenic response (41). However, S6K1-deficient mice are protected against diet-induced obesity (40).

PASK-deficient mice downregulate the expression of mRNAs encoding AMPKα2 and also modulate the GLP-1 effects in the hypothalamus (9); this suggests that both effects may be regulating thermogenesis in brown adipose tissue (BAT) and the browning of white fat, as both processes are mediated by the inhibition of hypothalamic AMPK (42, 43).

Peripheral and brain glucoregulatory systems cooperate to maintain glucose homeostasis and diabetes (44). In addition to acting in the hypothalamic functions, PASK also has key functions in the peripheral tissues. For example, diabetes and PASK have been linked, as a human mutation of the *PASK* gene has been correlated to the maturity-onset diabetes of the young (MODY). This mutation increases kinase activity and decreases glucose-stimulated insulin secretion by the pancreas (45). In addition, decreased PASK expression in pancreatic islets has been reported in human type 2 diabetics (6). The PASK function in peripheral tissues could be crucial for maintaining metabolic and energy homeostasis.

## Pask and the Nutritional Adaptation of the Liver

The first steps in understanding the role of PASK in liver metabolism involved analyzing the effect of an HFD in PASK-deficient mice, which recorded a reduced accumulation of liver triglyceride (5). However, these analyses also reported that this effect did not depend on changes in AMPK and S6K activities (5). The excessive accumulation of triglycerides in the liver in obesity has been related to the main regulator of lipogenesis, the sterol regulatory element binding protein (SREBP1c); a transcription factor that stimulates the expression of the enzymes involved in fatty acids and triglyceride synthesis (46, 47). PASK promotes the proteolytic maturation of SREBP1c (48); PASK-deficiency decreases the expression of the hepatic target genes of SREBP1c explaining the lower accumulation of triglyceride previously described (48).

An important finding in PASK’s role in controlling obesity is that its deficiency modifies the physiological response to fasting and refeeding (49, 50). One of the main hepatic functions is maintaining metabolic homeostasis during the adaptation to intermittent situations of food supply and fasting. This means that during fasting the liver plays a crucial role in maintaining blood glucose levels, initially through glycogenolysis, and subsequently through gluconeogenesis, while fatty acid oxidation provides part of the necessary energy. In the feeding condition, glucokinase facilitates glucose metabolism and the accumulation of its excess through the synthesis and storage of glycogen. In parallel, glycolytic pathway products are used to synthesize fatty acids and triglycerides, with both processes reflecting the liver’s role in quickly controlling glucose circulating levels after food intake. Although initial studies did not find a relationship between PASK and AMPK or S6K activity, subsequent studies have indicated that PASK deficiency alters the long fasting/feeding responses of AMPK and S6K in the liver. AMPK activity is higher under refeeding conditions than under fasting, and the activation level of S6K in this condition is significantly higher than in WT mice (9). The differences observed in the activation/inactivation status of both enzymes measured by phosphorylation degree might depend on the sample processing method in both studies, as we have previously reported the importance of inactivating phosphatases immediately to avoid variations over time (19).

**Figure 2:**
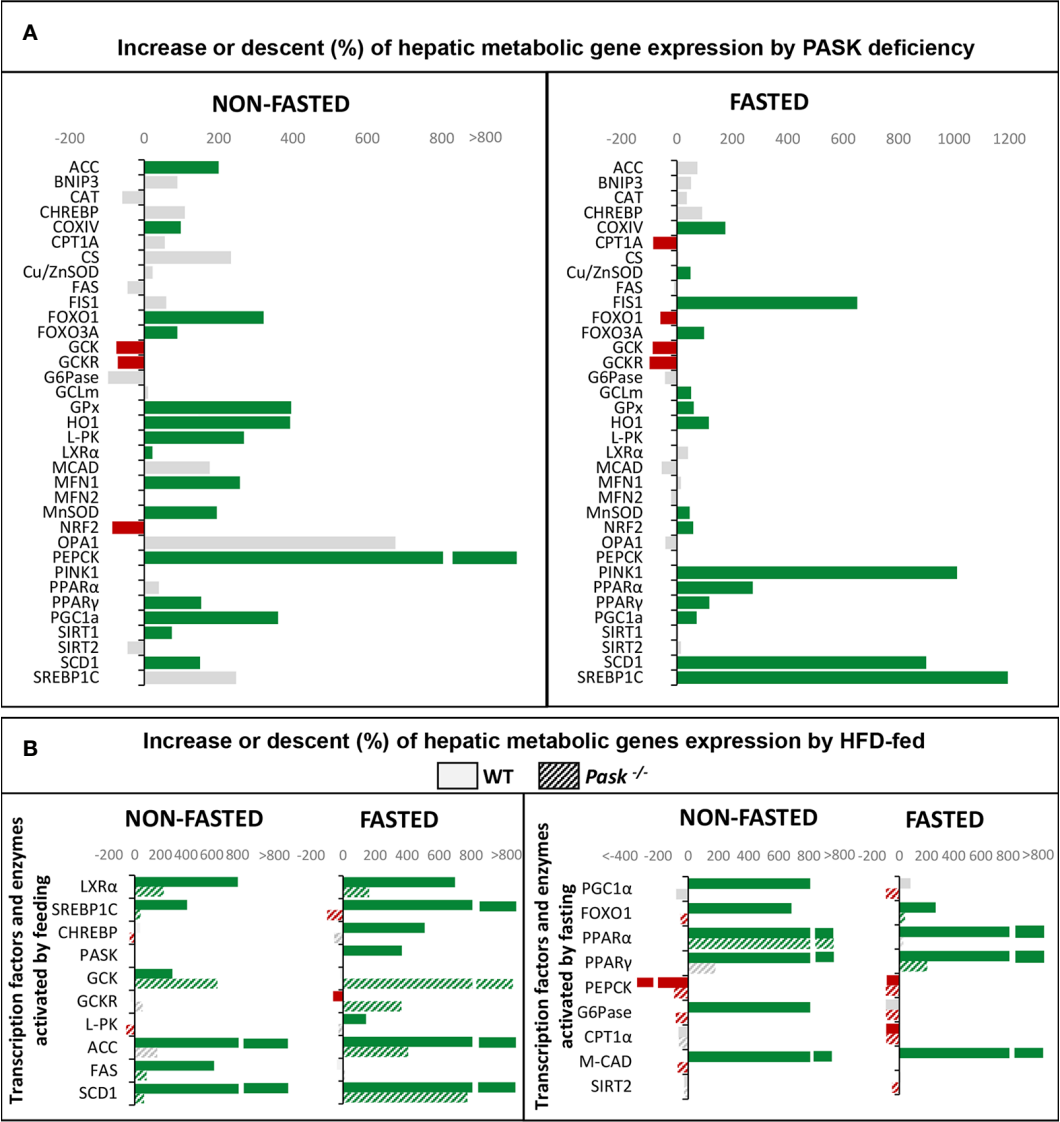
**(A)** Regulation of hepatic metabolic gene expression (mRNA) by Per-Arnt-Sim kinase (PASK) deficiency. Expression of genes (mRNA levels) that were upregulated (green) or downregulated (red) in PASK-deficient mice under fasting or non-fasting status. Grey represents non-significant variations. **(B)** Regulation of hepatic metabolic gene expression (mRNA) by a high-fat diet (HFD) in wild-type (WT) or PASK-deficient mice. Expression of transcription factors and enzymes that are usually upregulated by feeding (left panel) or by fasting (right panel). The bars represent upregulation (green) or downregulation (red) of mRNA levels under an HFD compared to a standard diet. Solid bars are data from WT mice. Dashed bars are data from PASK-deficient mice. Gray represents non-significant variations.

Our studies indicate that the role of PASK in hepatic metabolism is especially important in the adaptation to fasting/feeding states, where PASK deficiency influences the expression of several transcription factors and key enzymes in different metabolic pathways (49–52). **Figure 2A** therefore provides a summary of the more salient effects of PASK deficiency at transcriptional level observed in baseline conditions (non-fasted) and under fasting.

It is surprising how the inactivation of the kinase function in PASK modifies the expression of a significant number of transcription factors and key proteins of the main metabolic pathways responsible for maintaining homeostasis under fasting and feeding conditions.

PASK takes part in the complex regulatory mechanisms that facilitate the response to nutrient changes in the liver modulating the expression of several transcription factors and modifying the activities and stability of several enzymes involved in gluconeogenesis and mitochondrial fatty acid transport during fasting. For instance PASK deficiency modified the protein kinase B (AKT) activity, phosphoenolpyruvate carboxykinase (PEPCK) protein stability, and Carnitine Palmitoyltransferase 1A (CPT1A) gene expression altering its fasting response (50). Under feeding conditions in PASK-deficient mice, glucokinase activity is reduced together with a decrease in hepatic lipogenesis (50). Additionally, glycogen metabolism could also be modified because glycogen synthase is a PASK substrate (53). However, PASK deficiency does not cause detectable changes in the maintenance of blood glucose homeostasis during prolonged fasting periods (9).

PASK also plays an important role in conditions that generate some type of stress, such as fasting, which generate oxidative stress. In this situation, PASK regulates many mitochondrial functions in liver, from biogenesis to mitochondrial dynamics, playing a role in maintaining mitochondrial quality and antioxidant response mechanisms. **Figure 2A** shows the changes observed at transcriptional level of genes involved in hepatic oxidative stress metabolism and mitochondrial dynamics in PASK-deficient mice under non-fasting and fasting states (51). Likewise, PASK deficiency under fasting conditions induces the overexpression of antioxidant enzymes in liver, such as Glutamate-cysteine ligase (GCLm), Heme Oxygenase 1 (HO1), and proteins that modulate mitochondrial dynamics and mitophagy as Mitofusin 1/2 (MFN1/2), Optic atrophy 1 (OPA1), Mitochondrial receptor protein, Fission 1 (FIS1), BCL2 and adenovirus E1B 19-kDa-interacting protein (BNIP3) and Phosphatase and Tensin homolog (PTEN)-induced kinase 1 (PINK1) (51). The changes observed maintain ROS at steady levels and improve the regenerative state.

Another one of PASK’s main functions in the liver is to help avoid many of the deleterious effects produced by HFDs in liver metabolism to maintain metabolic homeostasis. **Figure 2B** summarizes some of the differences of expression of the transcription factors and enzymes that regulate the main metabolic pathways in PASK-deficient mice. The changes in those genes activated by feeding are shown in **Figure 2B**, left panel, and those activated during fasting in **Figure 2B**, right panel.

All the differences due to PASK deficiency lead to an improvement in glucose tolerance and insulin sensitivity, preventing weight gain and alterations in triglyceride and blood cholesterol values (49). The effects of pharmacologic PASK inhibition also confirm its role restoring insulin sensitivity and reducing the hepatic fat content and fibrosis associated with HFDs (54).

Although organisms are able to store calories and then consume them when they are needed, there are diets (e.g., HFDs) that induce disorders, altering all established regulatory mechanisms and causing obesity, insulin resistance, dyslipidemias, and alterations of caloric homeostasis. PASK’s roles at hepatic and central level contribute to caloric homeostasis. PASK deficiency does not cause detectable changes in the maintenance of blood glucose homeostasis at basal state during fasting or refeeding periods (9). Nevertheless, it is interesting to note that improved glucose tolerance and insulin sensitivity have been observed in HFD-fed PASK-deficient mice (49).

## Pask Epigenetics and Differentiation

PASK participates in the differentiation process of myogenic progenitor cells, embryonic stem cells, and adipogenic progenitor cells. PASK’s role is mediated by its substrate Wdr5, as described recently, which is part of the complexes that catalyze the methylation of the lysine 4 of histone H3. The modification of histone tails alters the chromatin structure and the accessibility of transcriptional machinery. This epigenetic modification thus regulates gene expression by promoting the repression/activation of gene promoters. PASK phosphorylates Wdr5, activating the trimethylation of the lysine 4 of histone H3 (H3K4me3) and the subsequent expression of myogenin, prompting muscle differentiation (55) (**Figure 1A**). PASK has been found to associate with the mammalian H3K4 MLL2 methyltransferase complex (56). PASK’s ability to regulate epigenetic modifications may explain how this protein kinase regulates the expression of a large number of genes. This PASK function also involves an interrelation with the other metabolic sensors, requiring the previous phosphorylation of PASK by the mTORC1 complex to initiate the initial stages of myogenesis, and the final stages of myogenesis require mTORC1-S6K signaling (57).

## Conclusions

PASK is a nutrient sensor whose expression responds to fasting/feeding in at least regulatory hypothalamic areas and the liver. Changes in PASK expression help the body to adapt to periods of fasting/feeding. Hypothalamic PASK contributes to the control of feeding and energy homeostasis. Hepatic PASK might mediate metabolic and caloric homeostasis during fasting/feeding cycles through the control of the expression of several transcription factors and key enzymes in the metabolic pathways activated or inhibited by nutritional changes. PASK’s role in epigenetic regulation may facilitate its ability to modify the expression of transcription factors and target enzymes in the main metabolic pathways: glycolysis, lipogenesis, and gluconeogenesis, which help the whole organism to adjust to periods of fasting/feeding for maintaining metabolic and caloric homeostasis.

## Author Contributions

VH-C, CS, and EA collected the data and previous results, analyzed, and updated the bibliography related to the topic. AP-G carried out the experimental procedures. CS plotted the data on the graphs. CS and EA wrote the manuscript, and all the authors revised the final version. All authors contributed to the article and approved the submitted version.

## Conflict of Interest

The authors declare that the research was conducted in the absence of any commercial or financial relationships that could be construed as a potential conflict of interest.
